# Supplementary material for: Lagging behind in health and work expectancies: Increasing disadvantage of individuals with lower educational level in Germany
Source: PLoS One. 2025 Nov 17;20(11):e0337064. doi: 10.1371/journal.pone.0337064 (PMC12622782; doi:10.1371/journal.pone.0337064)
Supplement: S1 File — (PDF) [file pone.0337064.s001.pdf]

Supplementary material

PlosOne

Lagging behind in health and work expectancies: Increasing disadvantage of individuals with lower educational level in Germany

Juliane Tetzlaff[1*], Fabian Tetzlaff[2], Marc Luy[3]

1 Medical Sociology Department, Hannover Medical School, Hannover, Germany
2 Division of Social Determinants of Health, Department of Epidemiology and Health Monitoring, Robert Koch Institute, Berlin, Germany
3 Vienna Institute of Demography of the Austrian Academy of Sciences, Vienna, Austria

*corresponding author:
Tetzlaff.Juliane@mh-hannover.de

Table S1 Partial Healthy Life Expectancy (HLE), Unhealthy Life Expectancy (UHLE), Healthy Working Life Expectancy (HWLE), and Unhealthy Working Life Expectancy (UHWLE) between ages 30 and 69 in terms of Physical Health-related Quality of Life by educational level, year, and gender

		Men							
**Education**		**HLE**	**95%-CI**	**UHLE**	**95%-CI**	**HWLE**	**95%-CI**	**UHWLE**	**95%-CI**
**lower**	2004	29.6	(29.1-30.1)	7.1	(6.7-7.5)	22.0	(21.5-22.6)	2.9	(2.6-3.2)
	2012	29.6	(29.1-30.1)	7.6	(7.1-8.1)	23.1	(22.5-23.7)	3.6	(3.3-4.0)
	2018	28.5	(27.8-29.1)	8.9	(8.3-9.6)	23.0	(22.2-23.8)	4.5	(3.9-5.0)
**middle**	2004	32.2	(31.5-32.9)	5.4	(4.7-6.0)	25.3	(24.5-26.1)	2.4	(2.0-2.8)
	2012	31.5	(30.8-32.1)	6.0	(5.4-6.6)	26.2	(25.5-26.9)	3.0	(2.6-3.4)
	2018	32.1	(31.4-32.8)	5.6	(5.0-6.2)	27.7	(27.0-28.4)	3.0	(2.6-3.4)
**higher**	2004	34.4	(34.0-34.9)	3.8	(3.4-4.3)	28.0	(27.5-28.6)	1.9	(1.6-2.1)
	2012	34.3	(33.9-34.7)	4.1	(3.7-4.6)	29.4	(28.9-29.9)	1.9	(1.7-2.2)
	2018	35.0	(34.5-35.4)	3.5	(3.1-3.9)	30.5	(29.9-31.1)	2.1	(1.7-2.4)
**total**	2004	31.5	(31.2-31.8)	5.9	(5.7-6.2)	24.5	(24.2-24.9)	2.5	(2.3-2.6)
	2012	31.7	(31.4-32.0)	6.1	(5.8-6.4)	26.1	(25.8-26.5)	2.9	(2.7-3.0)
	2018	32.0	(31.6-32.3)	6.0	(5.6-6.3)	27.2	(26.9-27.6)	3.1	(2.8-3.3)

		Women							
**Education**		**HLE**	**95%-CI**	**UHLE**	**95%-CI**	**HWLE**	**95%-CI**	**UHWLE**	**95%-CI**
**lower**	2004	30.0	(29.5-30.5)	8.4	(7.9-8.9)	16.8	(16.2-17.4)	2.9	(2.6-3.3)
	2012	29.2	(28.6-29.8)	9.1	(8.6-9.7)	18.7	(18.1-19.4)	3.8	(3.4-4.2)
	2018	28.2	(27.5-28.9)	10.1	(9.4-10.8)	19.3	(18.4-20.1)	4.5	(4.0-5.1)
**middle**	2004	32.4	(31.8-33.0)	6.3	(5.7-6.8)	20.4	(19.7-21.0)	2.4	(2.1-2.8)
	2012	31.8	(31.3-32.4)	6.9	(6.4-7.5)	22.9	(22.3-23.5)	3.2	(2.8-3.6)
	2018	31.6	(31.0-32.2)	7.2	(6.6-7.7)	24.8	(24.1-25.4)	3.6	(3.2-4.0)
**higher**	2004	33.3	(32.7-33.8)	5.8	(5.3-6.4)	22.4	(21.8-23.1)	2.5	(2.1-2.9)
	2012	33.8	(33.3-34.3)	5.4	(4.9-5.8)	25.4	(24.9-26.0)	2.8	(2.5-3.2)
	2018	33.8	(33.4-34.3)	5.4	(4.9-5.9)	26.8	(26.2-27.4)	3.2	(2.8-3.5)
**total**	2004	31.4	(31.1-31.7)	7.2	(6.9-7.5)	19.5	(19.1-19.8)	2.6	(2.4-2.8)
	2012	31.5	(31.2-31.8)	7.2	(7.0-7.5)	22.4	(22.1-22.7)	3.2	(3.0-3.4)
	2018	31.5	(31.2-31.8)	7.3	(7.0-7.6)	24.1	(23.7-24.5)	3.6	(3.4-3.9)

Health and Work Expectancies are given as partial life expectancies at age 30 up to age 69. 95% confidence intervals are given in brackets. Data: GSOEP 2000-2020, authors‘ own calculations, 95%-CI 95% confidence interval.

Table S2 Partial Healthy Life Expectancy (HLE), Unhealthy Life Expectancy (UHLE), Healthy Working Life Expectancy (HWLE), and Unhealthy Working Life Expectancy (UHWLE) between ages 30 and 69 in terms of Mental Health-related Quality of Life by educational level, year, and gender

					**Men**				
**Education**		**HLE**	**95%-CI**	**UHLE**	**95%-CI**	**HWLE**	**95%-CI**	**UHWLE**	**95%-CI**
**lower**	2004	31.1	(30.6-31.6)	5.6	(5.2-6.0)	21.8	(21.2-22.3)	3.2	(2.8-3.5)
	2012	31.2	(30.6-31.8)	6.1	(5.5-6.6)	23.1	(22.5-23.8)	3.6	(3.2-4.0)
	2018	31.8	(31.1-32.4)	5.7	(5.1-6.2)	24.5	(23.8-25.2)	3.0	(2.6-3.4)
**middle**	2004	32.5	(31.8-33.1)	5.1	(4.5-5.6)	24.1	(23.3-24.9)	3.6	(3.2-4.0)
	2012	32.2	(31.5-32.9)	5.3	(4.7-5.9)	25.3	(24.6-26.0)	3.9	(3.4-4.4)
	2018	33.3	(32.6-34.0)	4.4	(3.8-5.0)	27.6	(26.8-28.3)	3.1	(2.6-3.6)
**higher**	2004	32.5	(31.9-33.0)	5.8	(5.3-6.3)	25.6	(25.0-26.2)	4.3	(3.9-4.7)
	2012	33.8	(33.3-34.2)	4.7	(4.2-5.1)	27.8	(27.2-28.4)	3.5	(3.2-3.9)
	2018	34.1	(33.6-34.6)	4.3	(3.8-4.8)	29.2	(28.6-29.9)	3.3	(2.9-3.7)
**total**	2004	31.8	(31.5-32.1)	5.6	(5.3-5.9)	23.4	(23.0-23.7)	3.6	(3.4-3.8)
	2012	32.3	(32.0-32.7)	5.4	(5.1-5.7)	25.3	(24.9-25.7)	3.7	(3.4-3.9)
	2018	33.1	(32.7-33.4)	4.9	(4.5-5.2)	27.1	(26.7-27.5)	3.2	(2.9-3.4)
					**Women**				
**Education**		**HLE**	**95%-CI**	**UHLE**	**95%-CI**	**HWLE**	**95%-CI**	**UHWLE**	**95%-CI**
**lower**	2004	30.2	(29.6-30.8)	8.2	(7.7-8.7)	16.1	(15.5-16.7)	3.6	(3.3-4.0)
	2012	30.3	(29.7-31.0)	8.0	(7.4-8.6)	18.6	(17.9-19.3)	3.9	(3.5-4.3)
	2018	30.0	(29.3-30.7)	8.3	(7.6-9.0)	19.5	(18.7-20.4)	4.3	(3.7-4.8)
**middle**	2004	30.9	(30.2-31.5)	7.8	(7.3-8.4)	18.3	(17.6-18.9)	4.5	(4.1-4.9)
	2012	31.4	(30.8-31.9)	7.4	(6.9-8.0)	21.3	(20.7-21.9)	4.8	(4.4-5.2)
	2018	31.7	(31.1-32.3)	7.1	(6.5-7.7)	23.6	(22.9-24.3)	4.7	(4.2-5.3)
**higher**	2004	31.5	(30.9-32.1)	7.6	(7.1-8.2)	20.0	(19.3-20.7)	4.9	(4.5-5.4)
	2012	32.3	(31.8-32.8)	6.8	(6.3-7.4)	23.2	(22.6-23.8)	5.1	(4.6-5.5)
	2018	33.1	(32.7-33.6)	6.1	(5.6-6.6)	25.6	(25.0-26.2)	4.4	(4.0-4.8)
**total**	2004	30.7	(30.4-31.0)	7.9	(7.7-8.2)	17.8	(17.5-18.2)	4.3	(4.1-4.5)
	2012	31.3	(30.9-31.6)	7.5	(7.2-7.8)	21.0	(20.6-21.3)	4.6	(4.4-4.9)
	2018	31.9	(31.5-32.2)	6.9	(6.6-7.3)	23.3	(22.9-23.7)	4.4	(4.2-4.7)

Note: Health and Work Expectancies are given as partial life expectancies at age 30 up to age 69. 95% confidence intervals are given in brackets. Data: GSOEP 2000-2020, authors‘ own calculations, 95%-CI 95% confidence interval.

Table S3 Sensitivity Analysis using a PCS Cut-Off value of 50: Partial Healthy Life Expectancy (HLE), Unhealthy Life Expectancy (UHLE), Healthy Working Life Expectancy (HWLE), and Unhealthy Working Life Expectancy (UHWLE) between ages 30 and 69 in terms of Physical Health-related Quality of Life by educational level, year, and gender

		Men							
**Education**		**HLE**	**95%-CI**	**UHLE**	**95%-CI**	**HWLE**	**95%-CI**	**UHWLE**	**95%-CI**
**lower**	2004	18.7	(18.1-19.3)	18.0	(17.4-18.6)	14.7	(14.1-15.2)	10.3	(9.7-10.8)
	2012	18.0	(17.4-18.7)	19.2	(18.5-19.9)	14.9	(14.2-15.5)	10.4	(9.9-10.9)
	2018	17.4	(16.6-18.2)	20.1	(19.2-20.9)	14.7	(13.9-15.5)	12.8	(12.0-13.6)
**middle**	2004	21.5	(20.7-22.3)	16.1	(15.2-16.9)	18.0	(17.2-18.8)	9.7	(9.0-10.4)
	2012	20.3	(19.6-21.1)	17.2	(16.4-17.9)	17.8	(17.0-18.5)	11.5	(10.7-12.2)
	2018	20.4	(19.6-21.3)	17.3	(16.4-18.2)	18.5	(17.7-19.3)	12.2	(11.3-13.0)
**higher**	2004	25.5	(24.8-26.1)	12.8	(12.2-13.4)	21.8	(21.2-22.4)	8.1	(7.6-8.6)
	2012	25.4	(24.8-26.0)	13.0	(12.4-13.6)	22.5	(21.9-23.1)	8.8	(8.3-9.4)
	2018	25.9	(25.3-26.6)	12.5	(11.9-13.1)	23.2	(22.6-23.9)	9.3	(8.7-9.9)
**total**	2004	21.3	(21.0-21.7)	16.1	(15.7-16.4)	17.7	(17.3-18.0)	9.3	(9.0-9.6)
	2012	21.3	(20.9-21.7)	16.5	(16.1-46.9)	18.4	(18.1-18.8)	10.5	(10.2-10.9)
	2018	21.7	(21.3-22.2)	16.2	(15.8-16.6)	19.3	(18.9-19.7)	11.0	(10.6-11.4)

		Women							
**Education**		**HLE**	**95%-CI**	**UHLE**	**95%-CI**	**HWLE**	**95%-CI**	**UHWLE**	**95%-CI**
**lower**	2004	17.6	(17.0-18.2)	20.8	(20.1-21.4)	10.5	(9.9-11.0)	9.2	(8.7-9.8)
	2012	17.1	(16.4-17.8)	21.3	(20.5-22.0)	11.6	(10.9-12.2)	9.2	(8.7-9.8)
	2018	16.1	(15.4-16.9)	22.2	(21.3-23.0)	11.6	(10.8-12.4)	12.2	(11.5-13.0)
**middle**	2004	21.5	(20.8-22.2)	17.2	(16.5-17.9)	14.1	(13.5-14.7)	8.7	(8.2-9.3)
	2012	20.4	(19.7-21.1)	18.4	(17.7-19.0)	15.1	(14.5-15.7)	11.0	(10.5-11.6)
	2018	19.8	(19.1-20.5)	19.0	(18.2-19.7)	15.9	(15.2-16.6)	12.4	(11.8-13.1)
**higher**	2004	22.7	(22.0-23.4)	16.4	(15.7-17.1)	16.1	(15.4-16.7)	8.9	(8.3-9.5)
	2012	23.0	(22.4-23.6)	16.1	(15.5-16.8)	18.2	(17.6-18.8)	10.0	(9.5-10.6)
	2018	23.4	(22.8-24.1)	15.8	(15.2-16.5)	19.3	(18.7-19.9)	10.7	(10.1-11.3)
**total**	2004	20.1	(19.7-20.5)	18.5	(18.2-18.9)	13.2	(12.9-13.6)	8.9	(8.6-9.2)
	2012	20.2	(19.9-20.6)	18.5	(18.2-18.9)	15.1	(14.8-15.5)	10.4	(10.1-10.8)
	2018	20.2	(19.8-20.6)	18.6	(18.2-19.0)	16.1	(15.7-16.5)	11.6	(11.2-12.0)

Note: Health and Work Expectancies are given as partial life expectancies at age 30 up to age 69. 95% confidence intervals are given in brackets. Data: GSOEP 2000-2020, authors‘ own calculations, 95%-CI 95% confidence interval. Results presented in the paper (Figure 1 to 4) are based on a cut-off value of 40, which represents the threshold value of a meaningful deviation from the national PCS norm value of 50

Table S4 Sensitivity Analysis using a MCS Cut-Off value of 50: Partial Healthy Life Expectancy (HLE), Unhealthy Life Expectancy (UHLE), Healthy Working Life Expectancy (HWLE), and Unhealthy Working Life Expectancy (UHWLE) between ages 30 and 69 in terms of Mental Health-related Quality of Life by educational level, year, and gender

		Men							
**Education**		**HLE**	**95%-CI**	**UHLE**	**95%-CI**	**HWLE**	**95%-CI**	**UHWLE**	**95%-CI**
**lower**	2004	20.6	(20.0-21.2)	16.1	(15.6-16.7)	14.3	(13.8-14.9)	10.6	(10.1-11.1)
	2012	21.1	(20.4-21.8)	16.2	(15.5-16.9)	15.5	(14.9-16.2)	11.2	(10.6-11.8)
	2018	22.2	(21.4-23.0)	15.2	(14.4-16.0)	17.5	(16.6-18.3)	10.1	(9.3-10.8)
**middle**	2004	22.2	(21.4-23.1)	15.3	(14.5-16.1)	16.0	(15.2-16.7)	11.7	(11.0-12.4)
	2012	22.3	(21.5-32.2)	15.2	(14.4-16.0)	17.3	(16.5-18.0)	11.9	(11.2-12.7)
	2018	23.8	(22.9-24.8)	13.9	(13.0-14.8)	19.8	(18.9-20.7)	10.9	(10.1-11.7)
**higher**	2004	22.5	(21.8-23.2)	15.8	(15.1-16.5)	17.5	(16.9-18.1)	12.4	(11.8-13.0)
	2012	24.0	(23.3-24.7)	14.4	(13.8-15.1)	19.6	(18.9-20.3)	11.7	(11.1-12.3)
	2018	25.0	(24.3-25.7)	13.5	(12.8-14.1)	21.2	(20.5-21.9)	11.3	(10.7-12.0)
**total**	2004	21.4	(21.1-21.8)	16.0	(15.6-16.3)	15.6	(15.2-16.0)	11.4	(11.1-11.7)
	2012	22.4	(22.0-22.9)	15.3	(14.9-15.7)	17.4	(17.0-17.8)	11.6	(11.2-11.9)
	2018	23.7	(23.3-24.2)	14.2	(13.7-14.6)	19.6	(19.1-20.0)	10.7	(10.3-11.1)

		Women							
**Education**		**HLE**	**95%-CI**	**UHLE**	**95%-CI**	**HWLE**	**95%-CI**	**UHWLE**	**95%-CI**
**lower**	2004	18.7	(18.1-19.3)	19.7	(19.0-20.3)	10.1	(9.5-10.6)	9.6	(9.1-10.2)
	2012	19.1	(18.4-19.8)	19.3	(18.5-20.0)	11.7	(11.0-12.4)	10.8	(10.2-11.5)
	2018	19.5	(18.7-20.4)	18.8	(17.9-19.6)	13.1	(12.3-13.9)	10.7	(10.0-11.5)
**middle**	2004	19.8	(19.1-20.5)	18.9	(18.2-19.6)	11.4	(10.9-12.0)	11.4	(10.8-11.9)
	2012	21.0	(20.3-21.7)	17.8	(17.1-18.5)	14.0	(13.4-14.7)	12.1	(11.5-12.6)
	2018	22.1	(21.4-22.9)	16.6	(15.9-17.4)	16.2	(15.5-16.9)	12.1	(11.5-12.8)
**higher**	2004	20.3	(19.6-21.0)	18.8	(18.1-19.5)	12.6	(12.0-13.2)	12.3	(11.7-13.0)
	2012	21.4	(20.7-22.0)	17.8	(17.1-18.5)	15.0	(14.4-15.6)	13.3	(12.7-13.9)
	2018	22.9	(22.3-23.6)	16.3	(15.6-17.0)	17.7	(17.0-18.3)	12.3	(11.7-12.9)
**total**	2004	19.4	(19.0-19.7)	19.3	(18.9-19.6)	11.2	(10.8-11.5)	10.9	(10.6-11.3)
	2012	20.4	(20.0-20.8)	18.3	(18.0-18.7)	13.6	(13.2-13.9)	12.0	(11.7-12.4)
	2018	21.7	(21.3-22.2)	17.1	(16.7-17.5)	15.9	(15.5-16.3)	11.8	(11.4-12.2)

Note: Health and Work Expectancies are given as partial life expectancies at age 30 up to age 69. 95% confidence intervals are given in brackets. Data: GSOEP 2000-2020, authors‘ own calculations, 95%-CI 95% confidence interval. Results presented in the paper (Figure 1 to 4) are based on a cut-off value of 40, which represents the threshold value of a meaningful deviation from the national MCS norm value of 50

Figure S1 Time trends in Healthy, Unhealthy Life Expectancy, Healthy Working Life Expectancy, and Unhealthy Working Life Expectancy in terms of Physical Health−related Quality of Life at age 30 (to age 69) by year and gender

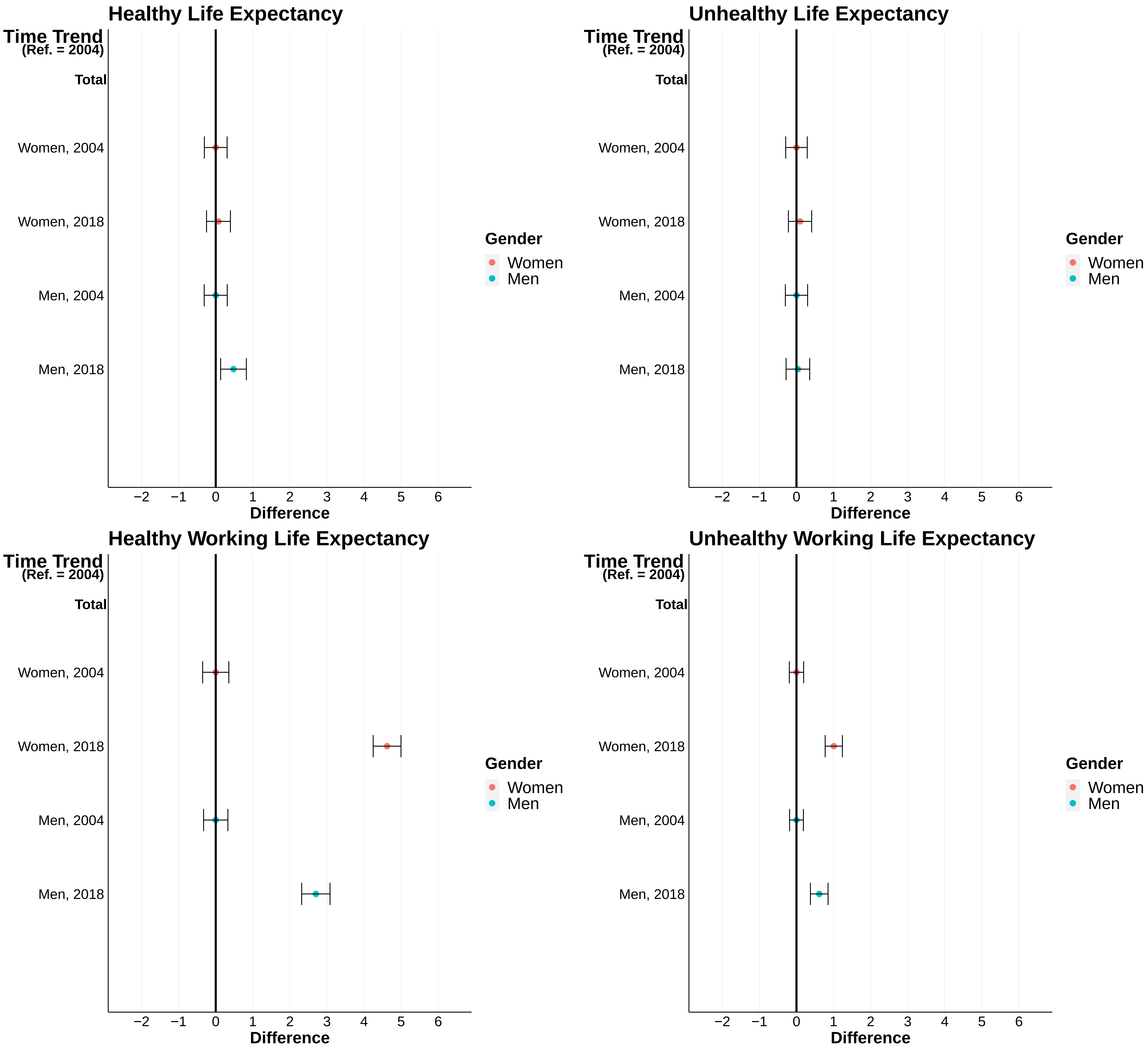

Note: Health and Work Expectancies are given as partial life expectancies at age 30 up to age 69. 95% confidence intervals are given in brackets. Data Source GSOEP 2000−2020, authors' own calculations. Data Source GSOEP 2000−2020, authors' own calculations.

Figure S2 Time trends in Healthy, Unhealthy Life Expectancy, Healthy Working Life Expectancy, and Unhealthy Working Life Expectancy in terms of Mental Health−related Quality of Life at age 30 (to age 69) by year and gender

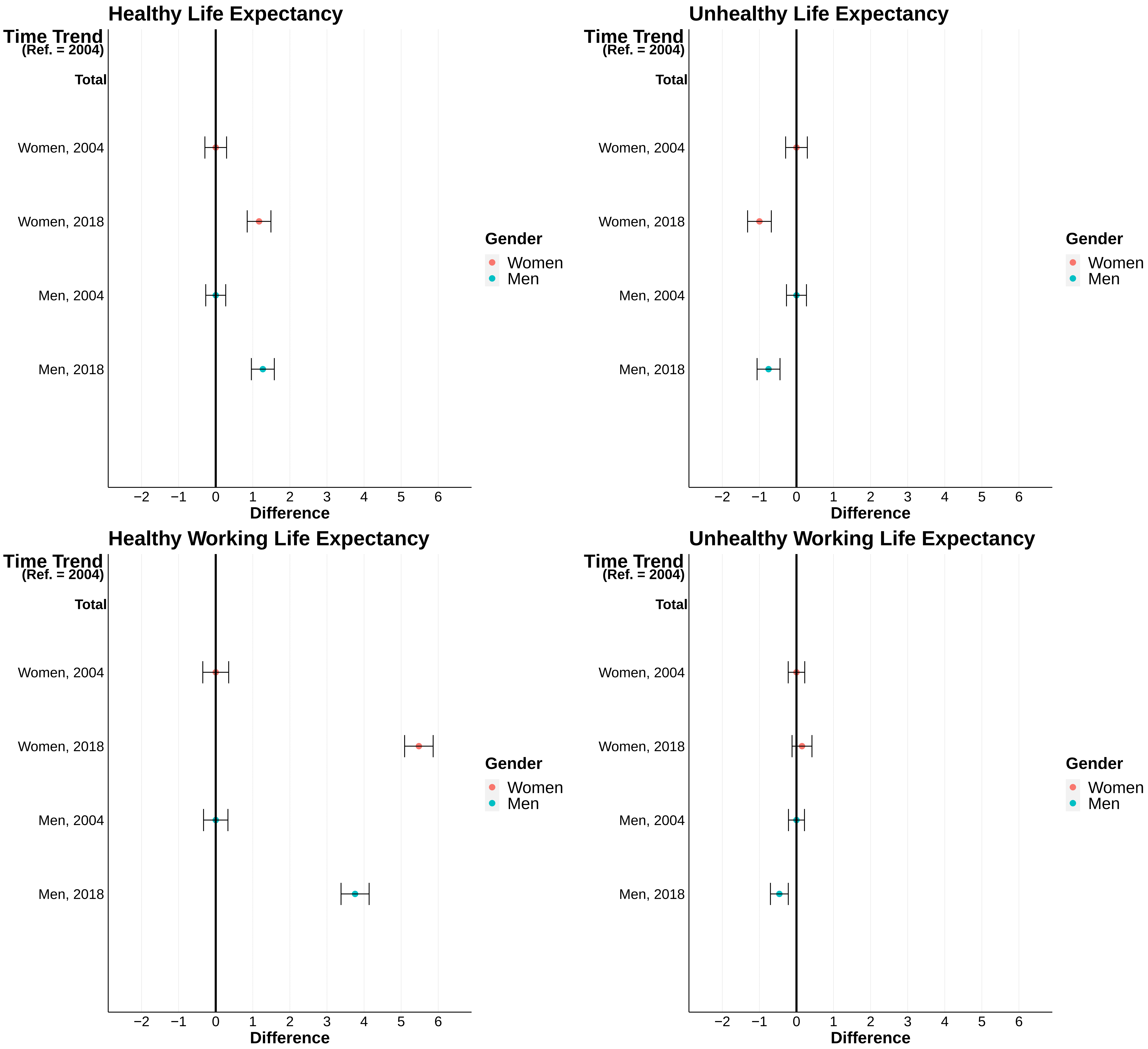

Note: Health and Work Expectancies are given as partial life expectancies at age 30 up to age 69. 95% confidence intervals are given in brackets. Data Source GSOEP 2000−2020, authors' own calculations. Data Source GSOEP 2000−2020, authors' own calculations.
